# Countrywide serological evaluation of canine prevalence for *Anaplasma* spp., *Borrelia burgdorferi* (*sensu lato*), *Dirofilaria immitis* and *Ehrlichia canis* in Mexico

**DOI:** 10.1186/s13071-016-1686-z

**Published:** 2016-07-29

**Authors:** Rebeca Movilla, Carlos García, Susanne Siebert, Xavier Roura

**Affiliations:** 1 Hospital Clínic Veterinari, Universitat Autònoma de Barcelona, Carrer de l’Hospital, 08193 Cerdanyola del Vallès, Barcelona, Spain; 2 Facultad de Medicina Veterinaria y Zootecnia, Facultad de Estudios Superiores Cuatitlán UNAM, Mexico Ciudad Universitaria, 04510 Mexico City D.F., Mexico; 3 Bayer Animal Health GmbH, 40789 Monheim and Marketing Companion Animal Products (CAP), InternationalBuilding 6210, 2.56, 51368 Leverkusen, Germany

**Keywords:** Dogs, Epidemiology, Canine Vector-Borne Diseases, In-Clinic ELISA Tests

## Abstract

**Background:**

Canine vector-borne diseases (CVBD) have become a major concern for canine and human public health. The aim of the study described here is to add epidemiological data regarding four pathogens responsible for CVBD, namely anaplasmosis, borreliosis, dirofilariosis and ehrlichiosis in a national survey conducted in Mexico.

**Methods:**

Seventy-four veterinary centres located in 21 federal Mexican states were asked to test dogs with clinical signs suspect for CVBD and healthy dogs, for detection of *Dirofilaria immitis* antigen and antibodies against *Anaplasma* spp., *Borrelia burgdorferi* (*sensu lato*) and *Ehrlichia canis* using the SNAP® 4DX® from IDEXX® Laboratories.

**Results:**

A total of 1706 dogs were tested, including 943 apparently healthy and 722 CVBD-suspect dogs. Infected dogs were 36.7 %. The highest percentages of infection with *E. canis* (51.0 %) and *Anaplasma* spp. (16.4 %) were obtained in the northwestern region, while *D. immitis* was most frequently found in the northeastern region of the country (8.9 %). Four dogs from the northwestern, northeastern, eastern and southeastern regions, respectively, were positive for *B. burgdorferi* (*sensu lato*). Northcentral regions showed lowest overall prevalence of infection (2.4 %). Co-infections were detected in 8.8 % of the dogs tested. Statistically significant lower positivity was found among dogs aged less than one year (23.2 %) and small-sized dogs (27.6 %), while higher prevalence of infection was found in dogs living outdoors (42.0 %), dogs with detectable tick infestation (43.3 %) and dogs that received treatment for tick-transmitted infections (58.8 %). Seropositivity was a risk factor for the presence of clinical signs as follows: *Anaplasma* spp. (OR = 2.63; 95 % CI: 1.88–3.67; *P* < 0.0001), *D. immitis* (OR = 2.52; 95 % CI: 1.61–3.95; *P* < 0.0001), *E. canis* (OR = 3.58; 95 % CI: 2.88–4.45; *P* < 0.0001), mixed infections (OR = 4.08; 95 % CI: 2.79–5.96; *P* < 0.0001), one or more agents (OR = 3.58; 95 % CI: 2.91–4.42; *P* < 0.0001).

**Conclusions:**

Canine serological evidence supports that dogs from Mexico are at risk of acquiring *Anaplasma* spp., *D. immitis* and/or *E. canis*, while *B. burgdorferi* (*sensu lato*) transmission is minimal in the country. Practitioners play a fundamental role in the detection and control of these diseases to protect dogs and humans.

**Electronic supplementary material:**

The online version of this article (doi:10.1186/s13071-016-1686-z) contains supplementary material, which is available to authorized users.

## Background

In the last decades, worldwide emergence and reemergence of many canine vector-borne diseases (CVBD) have been documented [1–4]. A diverse range of pathogens transmitted by haematophagous arthropods, mainly ticks and mosquitoes, cause CVBD [5], which have become a major focus of interest due to their importance to canine and human public health. In addition to suffering from illness, dogs can be subclinically infected and act as reservoir hosts for arthropod-transmitted zoonotic pathogens [6]. The increasing canine population and the close relationship with humans in both rural and urban areas add new concerns [7].

Anaplasmosis, borreliosis, dirofilariosis and ehrlichiosis have been recognized as some of the major CVBD with global significance [8].

*Anaplasma phagocytophilum* is a Gram-negative obligate intracellular bacterium, transmitted by ticks of the genus *Ixodes. Anaplasma phagocytophilum* infects granulocytes, mainly neutrophils, causing granulocytic anaplasmosis in mammalian hosts, including dogs and humans [9]. *Anaplasma platys,* transmitted by *R. sanguineus* (*sensu lato*) infects canine platelets and is responsible for the infectious canine cyclic thrombocytopenia. Pathogenicity is generally low but *A. platys* may play a role in co-infection with other arthropod-borne diseases [10]. The close molecular relationship between *A. phagocytophilum* and *A. platys* limits the serological differentiation between both agents due to cross-reactions [11].

*Borrelia burgdorferi* (*sensu lato*) is the causative agent of Lyme disease or borreliosis in mammalian hosts. Ticks of the genus *Ixodes* are the main vectors of this spirochete [12]. Dogs are susceptible to infection but clinical disease generally is milder and less frequent than in humans [13]. Because of their frequent exposure to ticks and ready seroconversion, dogs have been proposed as sentinels for risk of Lyme disease in humans [14].

Cardiopulmonary dirofilariosis is a potentially fatal disease caused by infection mainly with the adult stages of the nematode *Dirofilaria immitis*, also known as heartworm, and mosquitoes of the genus *Aedes* and *Culex* are considered main vectors [15]. Human heartworm infection is incidental and typically not associated with severe clinical signs. However, human cases have been reported in areas of high canine prevalence, highlighting the importance of heartworm testing and chemoprophylaxis in dogs to reduce transmission [16].

The Gram-negative bacterium *Ehrlichia canis* is the causative agent of canine monocytic ehrlichiosis, transmitted worldwide by the brown dog tick *Rhipicephalus sanguineus* (*sensu lato*) [17, 18]. Diagnosis is challenging due to its different phases and multiple clinical-pathological manifestations [19, 20].

Prevalence of CVBD is dependent on favourable climate and habitat for the microbiologic pathogens, their arthropod vectors and their mammalian reservoirs [2]. Mexico has a great diversity of climates determined by several factors such as the altitude, geographical latitude, weather conditions and the existing distribution of land and water [21]. The existence of favourable environments for the development of CVBD across the country has already been demonstrated, but knowledge of the prevalence and distribution of CVBD in Mexico is scarce and most data rely on geographically limited studies and/or the assessment of a low number of pathogens [22–31].

Epidemiological tools are essential to guide the definitive etiological diagnosis of CVBD and establish adequate methods for prevention [32]. Studies mapping the national seroprevalence of certain CVBD agents have been recently conducted in other countries [33–49]. However, to the authors' knowledge, this is the first description in Mexico at the countrywide level.

This study aimed to add useful information about the national serological distribution and epidemiological associations of four important vector-borne pathogens among dogs from different Mexican states.

## Methods

### Source of data

**Fig. 1:**
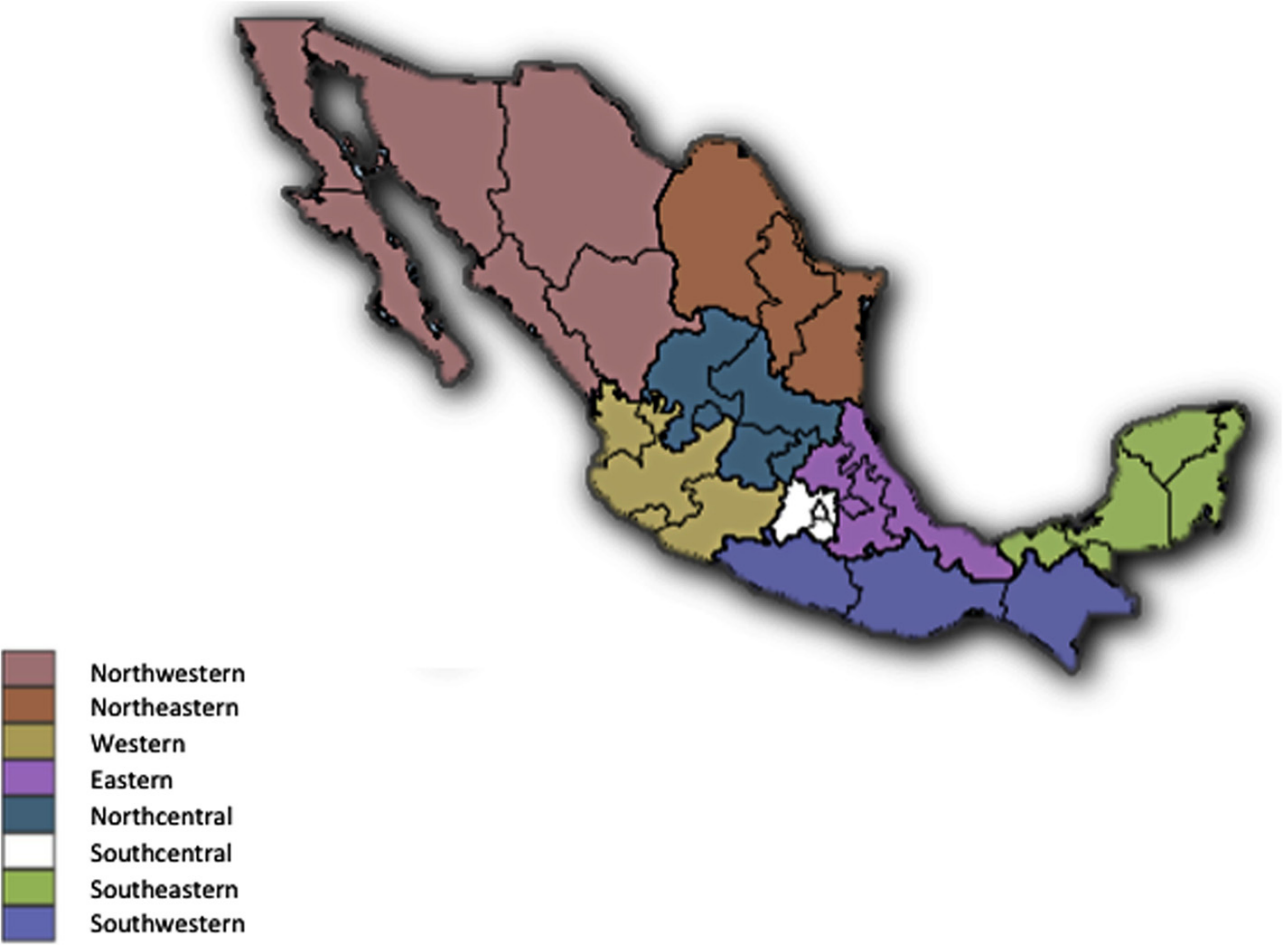
Geoeconomic regions of Mexico. Distribution of the federated states of Mexico, according to a geoeconomic division, into eight different geoeconomic regions as follows: northwestern, northeastern, western, eastern, northcentral, southcentral,southeastern and southwestern

Seventy-four veterinary centres located in 28 cities represented 21 out of 32 Federate Mexican states in this study. Cities were selected in locations where CVBD agents are more likely to be found. In order to geographically assess canine seroprevalence, states were grouped into eight different geoeconomic regions: northwestern (Baja California Norte, Baja California Sur, Chihuahua, Sinaloa and Sonora), northeastern (Coahuila and Nuevo Leon), western (Colima, Jalisco and Nayarit), eastern (Puebla, Veracruz and Hidalgo), northcentral (Aguascalientes, Guanajuato and Querétaro), southcentral (Morelos), southeastern (Quintana Roo and Yucatán) and southwestern (Chiapas and Guerrero) (Fig. 1).

Each veterinary centre received at least 30 in-clinic tests. Veterinarians were asked to test dogs with clinical signs suspect for anaplasmosis, borreliosis, dirofilariosis and/or ehrlichiosis, and a similar number of apparently healthy dogs, under physical examination, from April 2011 to January 2012. Inclusion criteria for testing sick dogs was considered for the presence of one or more of the following clinical alterations: lethargy, hyperthermia, weakness, weight loss, oedema, polyuria-polydipsia, lymph node enlargement, joint inflammation, lameness, cough, dyspnoea, nasal discharge, pale mucous membranes, haemorrhagic disorders, ocular lesions or neurologic signs. The absence of consistent history and/or evidence of abnormalities compatible with CVBD were considered exclusion criteria and these dogs were included in the healthy group.

The study was carried out in accordance with the International Guiding Principles for Biomedical Research Involving Animals, issued by the Council for the International Organizations of Medical Sciences. Dogs were entered in the study under owner consent. Veterinarians recorded additional data of the dogs using a questionnaire that included health status, gender, age (< 1 year or ≥ 1 year), size [small (< 10 kg), medium (10–25 kg) or large (> 25 kg)], lifestyle (outdoors or indoors), detectable tick infestation, use of treatment (mainly tetracycline, doxycycline, imidocarb and/or ivermectin) against tick and mosquito-transmitted infections as those evaluated here, and travel history to other regions or abroad.

### Serological testing

Whole blood, serum or plasma samples from dogs were tested by means of a rapid in-clinic enzyme-linked immunosorbent assay (ELISA) kit (SNAP® 4Dx® from IDEXX® Laboratories, Westbrook, Maine, USA), according to the instructions listed in the product package insert. This assay screens for simultaneous qualitative detection of a circulating carbohydrate of *D. immitis* antigen, from the adult female heartworms, and antibodies, immunoglobulin G and M, against immunodominant proteins of *A. phagocytophylum* (p44/MSP2), *B. burgdorferi* (*sensu lato*) (C6) and *E. canis* (p30 and p30-1)*.* Preliminary studies indicate that *A. phagocytophilum* analyte in SNAP® 4Dx® cross-reacts with samples from *A. platys-*infected dogs (SNAP® 4Dx® kit insert, unpublished data). Reported sensitivity and specificity of the in-clinic ELISA for detection of antibodies are 99.1 and 100 % for *A. phagocytophilum*, 98.8 and 100 % for *B. burgdorferi* (*sensu lato*) and 96.2 and 100 % for *E. canis*. Reported sensitivity and specificity for detection of heartworm antigen were 99.2 and 100 %, respectively [50].

### Data analysis

Statistical analysis was performed using SPSS 20.0 software. Values for prevalence were established. Chi-square and Fisher’s exact tests were used to compare proportions of positivity related to categorical dependent variables. A probability *P*-value  < 0.05 was considered statistically significant. Logistic regression analysis was subsequently performed. Significant differences between categories were quantified calculating odds ratios (OR) and their 95 % confidence intervals (CI).

## Results

Practitioners tested a total of 1706 dogs over the study period. Information about health status was recorded for 1665 dogs: 943 apparently healthy and 722 CVBD-suspect dogs; no data were available for 41 dogs. Table 1 summarizes the characteristics of the population.

**Table 1:** Population description

Characteristic	Health status			Total population
	Healthy dogs	CVBD-suspect dogs	Not known	
Gender				
Male	486	392	23	901
Female	442	322	17	781
Not known	15	8	1	24
Age				
< 1 year	41	36	5	82
≥ 1 year	874	667	29	1570
Not known	28	19	7	54
Median age	4 years	5 years	5 years	5 years
	IQR: 2–7	IQR: 3–8	IQR: 3–9	IQR: 3–8
Size				
Small (< 10 kg)	383	234	14	631
Medium (10–25 kg)	103	121	2	226
Large (> 25 kg)	304	200	18	522
Not known	153	167	7	327
Lifestyle				
Indoors	269	166	12	447
Outdoors	620	516	23	1159
Not known	54	40	6	100
Tick contact				
Yes	627	602	29	1258
No	268	68	5	341
Not known	48	52	7	107
TTI prevention				
Yes	46	36	3	85
No	890	676	27	1593
Not known	7	10	11	28
MTI prevention				
Yes	43	42	0	85
No	891	667	27	1585
Not known	9	13	14	36
Travel history				
Yes	176	151	4	331
No	763	569	33	1365
Not known	4	2	4	10

*Abbreviations*: *IQR* interquartile range, *TTI* tick-transmitted infections, *MTI* mosquito-transmitted infections

Overall and regional seroprevalence of all the dogs included in the study, apparently healthy dogs and CVBD-suspect dogs are shown in Tables 2, 3 and 4.

**Table 2:** Geographical distribution of seroprevalence to CVBD agents among all the dogs included in the study

Region	Dogs	Dogs infected (%) [95 % CI]	Positivity (%) [95 % CI]				Dogs co-infected (%) [95 % CI]
			*A*	*Bb*	*Di*	*Ec*	
Northwestern	384	213 (55.47)^**^	63 (16.4)^**^	1 (0.26)	25 (6.51)	196 (51.04)^**^	64 (16.67)^**^
		[50.3–60.2]	[12.8–20.1]	[0.0–0.8]	[4.2–9.4]	[46.1–56.0]	[12.5–20.3]
Northeastern	349	114 (32.66)	23 (6.59)^*^	1 (0.29)	31 (8.88)^*^	80 (22.9)^**^	19 (5.44)^*^
		[27.8–37.5]	[4.3–9.2]	[0.0–0.8]	[6.0–12.0]	[18.6–27.5]	[3.4–8.0]
Northcentral	164	4 (2.44)^**^	1 (0.61)^**^	0 (0)	0 (0)^*^	4 (2.43)^**^	1 (0.61)^**^
		[0.6–4.9]	[0.0–1.8]	[0.0–0.0]	[0.0–0.0]	[0.6–4.9]	[0.0–1.8]
Western	140	54 (38.57)	14 (10)	0 (0)	3 (2.14)	52 (37.14)	15 (10.71)
		[30.5–46.4]	[5.7–15.0]	[0.0–0.0]	[0.0–5.0]	[28.6–45.0]	[5.7–15.7]
Eastern	205	54 (26.34)^*^	18 (8.78)	1 (0.49)	10 (4.88)	37 (18.05)^**^	11 (5.37)
		[20.5–32.2]	[4.9–12.7]	[0.0–1.5]	[2.0–7.8]	[13.0–23.4]	[2.4–8.8]
Southcentral	86	17 (19.77)^*^	4 (4.65)	0 (0)	2 (2.32)	13 (15.12)^*^	2 (2.32)^*^
		[11.6–29.1]	[1.2–9.3]	[0.0–0.0]	[0.0–5.8]	[8.1–23.3]	[0.0–5.8]
Southwestern	149	67 (44.97)^*^	19 (12.75)	0 (0)	2 (1.34)^*^	63 (42.3)^*^	16 (10.74)
		[36.9–53.0]	[7.4–18.8]	[0.0–0.0]	[0.0–3.4]	[34.2–50.3]	[6.0–16.1]
Southeastern	229	103 (44.98)^*^	27 (11.79)	1 (0.44)	18 (7.86)	81 (35.37)	23 (10.04)
		[38.4–51.5]	[7.4–16.2]	[0.0–1.3]	[4.8–11.8]	[29.3–41.5]	[6.6–14.0]
Mexican states	1706	626 (36.7)	169 (9.91)	4 (0.23)	91 (5.33)	526 (30.83)	151 (8.85)
		[34.3–39.0]	[8.6–11.4]	[0.1–0.5]	[4.3–6.4]	[28.7–33.1]	[7.5–10.2]

*Abbreviations*: *A* *Anaplasma* spp., *Bb* *Borrelia burgdorferi* (*sensu lato*), *Di* *Dirofilaria immitis*, *Ec* *Ehrlichia canis*

* Statistically significant difference (*P* < 0.05); ** Statistically significant difference (*P* < 0.0001) to the corresponding national average value of prevalence

**Table 3:** Geographical distribution of seroprevalence to CVBD agents among apparently healthy dogs

Apparently healthy dogs							
Region	Dogs	Dogs infected (%) [95 % CI]	Positivity (%) [95 % CI]				Dogs co-infected (%) [95 % CI]
			*A*	*Bb*	*Di*	*Ec*	
Northwestern	177	62 (35.03)^**^	22 (12.43)^**^	0 (0)	10 (5.65)	55 (31.07)^**^	21 (11.86)^**^
		[28.2–41.8]	[7.9–17.5]	[0.0–0.0]	[2.8–9.0]	[24.7–37.9]	[7.3–16.9]
Northeastern	223	48 (21.52)	6 (2.69)^*^	0 (0)	13 (5.83)^*^	33 (14.79)	4 (1.79)
		[16.6–26.9]	[0.9–4.9]	[0.0–0.0]	[3.1–9.0]	[10.3–19.4]	[0.4–3.6]
Northcentral	107	0 (0)^**^	0 (0)^*^	0 (0)	0 (0)^*^	0 (0)^**^	0 (0)^*^
		[0.0–0.0]	[0.0–0.0]	[0.0–0.0]	[0.0–0.0]	[0.0–0.0]	[0.0–0.0]
Western	57	19 (33.33)	4 (7.02)	0 (0)	2 (3.51)	17 (29.82)	4 (7.02)
		[21.1–45.6]	[1.8–14.0]	[0.0–0.0]	[0.0–8.8]	[17.5–42.1]	[1.8–14.0]
Eastern	131	26 (19.85)	7 (5.34)	0 (0)	5 (3.82)	17 (12.98)	2 (1.53)
		[13.0–27.5]	[1.5–9.2]	[0.0–0.0]	[0.8–7.6]	[7.6–19.1]	[0.0–3.8]
Southcentral	46	5 (10.87)^*^	2 (4.35)	0 (0)	0 (0)	4 (8.69)	1 (2.17)
		[2.2–20.3]	[0.0–10.9]	[0.0–0.0]	[0.0–0.0]	[2.2–17.4]	[0.0–6.5]
Southwestern	86	34 (39.53)^*^	5 (5.81)	0 (0)	0 (0)	32 (37.21)^**^	3 (3.49)
		[29.1–50.0]	[1.2–11.6]	[0.0–0.0]	[0.0–0.0]	[27.5–47.7]	[0.0–8.1]
South-eastern	116	33 (28.45)	12 (10.35)	0 (0)	1 (0.86)	24 (20.69)	4 (3.45)
		[20.7–37.1]	[5.2–16.4]	[0.0–0.0]	[0.0–2.6]	[13.8–28.4]	[0.9–6.9]
Mexican states	943	227 (24.07)	58 (6.15)	0 (0)	31 (3.29)	182 (19.30)	39 (4.13)
		[21.3–26.8]	[4.7–7.6]	[0.0–0.0]	[2.2–4.5]	[16.8–21.8]	[3.0–5.5]

*Abbreviations*: *A Anaplasma* spp., *Bb* *Borrelia burgdorferi* (*sensu lato*), *Di* *Dirofilaria immitis*, *Ec* *Ehrlichia canis*

* Statistically significant difference (*P* < 0.05); ** Statistically significant difference (*P* < 0.0001) to the corresponding national average value of prevalence

**Table 4:** Geographical distribution of seroprevalence to CVBD agents among dogs with clinical signs

CVBD-suspect dogs							
Region	Dogs	Dogs infected (%) [95 % CI]	Positivity (%) [95 % CI]				Dogs co-infected (%) [95 % CI]
			*A*	*Bb*	*Di*	*Ec*	
Northwestern	203	149 (73.39)^**^	41 (20.19)^*^	1 (0.49)	14 (6.89)	140 (68.96)^**^	43 (21.18)^*^
		[66.5–78.8]	[14.8–25.6]	[0.0–01.5]	[0.0–0.0]	[62.6–74.9]	[14.8–26.3]
Northeastern	113	59 (52.21)	14 (12.39)	1 (0.88)	16 (14.16)^*^	43 (38.05)	13 (11.50)
		[42.5–61.1]	[6.2–19.5]	[0.0–2.7]	[8.0–20.4]	[29.2–47.8]	[5.3–17.7]
Northcentral	55	4 (7.27)^**^	1 (1.81)^*^	0 (0)	0 (0)^*^	4 (7.27)^**^	1 (1.81)^*^
		[1.8–14.5]	[0.0–5.5]	[0.0–0.0]	[0.0–0.0]	[1.8–14.5]	[0.0–5.5]
Western	82	35 (42.68)^*^	10 (12.19)	0 (0)	1 (1.22)^*^	35 (42.68)	11 (13.41)
		[32.9–53.7]	[6.1–19.5]	[0.0–0.0]	[0.0–3.7]	[31.7–52.4]	[6.1–20.7]
Eastern	70	28 (40.0)^*^	11 (15.71)	1 (1.43)	5 (7.14)	20 (28.57)^*^	9 (12.86)
		[28.6–51.4]	[7.1–24.3]	[0.0–4.3]	[1.4–12.9]	[18.6–40.0]	[5.7–21.4]
Southcentral	39	12 (30.77)^*^	2 (5.13)	0 (0)	2 (5.13)	9 (23.08)^*^	1 (2.56)^*^
		[17.1–46.2]	[0.0–12.8]	[0.0–0.0]	[0.0–12.8]	[10.3–35.9]	[0.0–7.7]
Southwestern	53	27 (50.94)	12 (22.64)	0 (0)	2 (3.77)	25 (47.17)	11 (20.75)
		[37.7–64.2]	[11.3–35.8]	[0.0–0.0]	[0.0–9.4]	[34.0–60.4]	[11.3–32.1]
Southeastern	107	70 (65.42)^*^	15 (14.01)	1 (0.93)	17 (15.89)^*^	57 (53.27)	19 (17.76)
		[56.1–73.8]	[7.5–20.6]	[0.0–3.1]	[9.3–23.4]	[43.0–62.6]	[10.9–25.2]
Mexican states	722	384 (53.18)	106 (14.68)	4 (0.56)	57 (7.89)	333 (46.12)	108 (14.96)
		[49.5–56.9]	[12.3–17.3]	[0.1–1.1]	[6.0–10.0]	[42.4–49.7]	[12.3–17.6]

*Abbreviations*: *A Anaplasma* spp., *Bb* *Borrelia burgdorferi* (*sensu lato*), *Di* *Dirofilaria immitis*, *Ec* *Ehrlichia canis*

* Statistically significant difference (*P* < 0.05); ** Statistically significant difference (*P* < 0.0001) to the corresponding national average value of prevalence

Of the 1706 dogs tested, 626 (36.7 %) were positive to one or more agents. The highest prevalence was detected in the Northwest (55.5 %), where seropositivity to *E. canis* (51.0 %) and *Anaplasma* spp. (16.4 %) was significantly higher than in the remaining regions. *Dirofilaria immitis* was more frequently detected in the Northeast (8.9 %). However, seropositivity to *B. burgdorferi* (*sensu lato*) was similar in the eight regions. Lowest seroprevalence was observed in the northcentral area of the country (2.4 %).

Table 5 describes positive results for more than one agent in 151 of 1706 dogs (8.8 %), mostly between *E. canis* and *Anaplasma* spp. No dog was positive for all agents.

**Table 5:** Positivity to single agents and co-infections among all dogs included in the study, apparently healthy and CVBD-suspect dogs

Pathogens	Healthy dogs		CVBD-suspect dogs		Total population	
	*n*	%	*n*	%	*n*	%
Single agent	188	19.9^*^	276	38.2^*^	475	27.84
*A*	27	2.9	22	3.0	50	2.93
*Bb*	0	0	2	0.3	2	0.12
*Di*	16	1.7	23	3.2	42	2.46
*Ec*	145	15.4^*^	229	31.7^*^	381	22.33
Co-infections	39	4.1^*^	108	15^*^	151	8.85
*Ec* + *Di*	8	0.8^*^	23	3.2^*^	31	1.82
*Ec* + *Bb*	0	0	1	0.1	1	0.06
*Ec* + *A*	24	2.5^*^	72	10^*^	100	5.86
*Di* + *Bb*	0	0	0	0	0	0
*Di* + *A*	2	0.2	4	0.6	6	0.35
*Bb* + *A*	0	0	0	0	0	0
*Ec* + *Di* + *Bb*	0	0	0	0	0	0
*Ec* + *Di* + *A*	5	0.5	7	1.0	12	0.7
*Ec* + *Bb* + *A*	0	0	1	0.1	1	0.06
*Di* + *Bb* + *A*	0	0	0	0	0	0
*Ec* + *Di* + *Bb* + *A*	0	0	0	0	0	0

*Abbreviations*: *A Anaplasma* spp., *Bb B. burgdorferi* (*sensu lato*), *Di D. immitis*, Ec *E. canis*

^*^Statistically significant difference (*P* < 0.05)

Seropositivity to the different CVBD agents according to the variables analysed is shown in Table 6. Odds ratios (OR) and 95 % CI for binomial variables presenting significant associations are summarized in Table 7. For all of the dogs examined no association was observed between gender and any CVBD agent. Overall, dogs aged 1 year-old or older presented a higher prevalence (37.0 %) than dogs aged less than 1 year (23.2 %), *χ*^*2*^ = 6.450, *df* = 1, *P* < 0.011. Positivity among small-sized dogs (27.6 %) was significantly lower than positivity for medium (43.4 %) and large breed dogs (39.8 %), *χ*^*2*^ = 27.676, *df* = 2, *P* < 0.001. Prevalence among dogs living outdoors was higher than indoors (42.0 and 23.7 %, respectively; *χ*^*2*^ = 46.412, *df* = 1, *P* < 0.001). Dogs with detectable tick contact were more frequently positive to CVBD agents (43.3 %) than dogs without ticks (9.7 %), *χ*^*2*^ = 131.577, *df* = 1, *P* < 0.001. In addition, higher prevalence was detected among dogs that received treatment for tick-transmitted infections than dogs that did not (58.8 and 35.6 %, respectively; *χ*^*2*^ = 18.730, *df* = 1, *P* < 0.001), while receiving treatment for mosquito-transmitted infections was not linked to the presence of CVBD agents. No relationship was detected between positivity and previous travel history. Seropositivity was a risk factor for the presence of clinical signs (*χ*^*2*^ = 149.200, *df* = 1, *P* < 0.001).

**Table 6:** Seropositivity for CVBD agents according to the epidemiological variables analysed

Epidemiological variable (*n*)	Total population seropositivity (%)					
	Dogs infected (%)	*A*	*Bb*	*Di*	*Ec*	Dogs co-infected (%)
Gender	*P* = 0.205	*P* = 0.871	*P* = 0.886	*P* = 0.772	*P* = 0.291	*P* = 0.565
Male (901)	343 (38.07)	89 (9.88)	2 (0.22)	49 (5.44)	288 (31.96)	77 (8.55)
Female (781)	274 (35.08)	79 (10.11)	2 (0.26)	40 (5.12)	231 (29.58)	73 (9.35)
Age	*P* = 0.011	*P* = 0.958	*P* = 0.647	*P* = 0.096	*P* = 0.014	*P* = 0.379
< 1 year (82)	19 (23.17)	8 (9.76)	0 (0)	1 (1.22)	15 (18.29)	5 (6.09)
≥ 1 year (1570)	581 (37.01)	156 (9.94)	4 (0.25)	85 (5.41)	489 (31.14)	140 (8.92)
Size	*P* < 0.0001	*P* = 0.606	*P* = 0.542	*P* = 0.001	*P* = 0.047	*P* < 0.0001
Small (631)	174 (27.58)	44 (6.97)	0 (0)	13 (2.06)	146 (23.14)	27 (4.28)
Medium (226)	98 (43.36)	25 (11.06)	1 (0.44)	17 (7.52)	80 (35.39)	23 (10.18)
Large (522)	208 (39.85)	55 (10.54)	2 (0.38)	36 (6.89)	172 (32.95)	51 (9.77)
Lifestyle	*P* < 0.0001	*P* = 0.001	*P* = 0.214	*P* < 0.0001	*P* < 0.0001	*P* < 0.0001
Indoors (447)	106 (23.71)	27 (6.04)	0 (0)	8 (1.79)	88 (19.69)	17 (3.80)
Outdoors (1159)	487 (42.02)	133 (11.47)	4 (0.34)	81 (6.99)	407 (35.12)	125 (10.78)
Tick contact	*P* < 0.0001	*P* < 0.0001	*P* = 0.297	*P* = 0.002	*P* < 0.0001	*P* < 0.0001
Yes (1258)	545 (43.32)	156 (12.40)	4 (0.32)	80 (6.36)	458 (36.40)	141 (11.21)
No (341)	33 (9.68)	4 (1.17)	0 (0.00)	7 (2.05)	25 (7.33)	3 (0.88)
TTI prevention	*P* < 0.0001	*P* = 0.188	*P* = 0.069	*P* = 0.429	*P* < 0.0001	*P* = 0.033
Yes (85)	50 (58.82)	12 (14.12)	1 (1.18)	3 (3.53)	49 (57.65)	13 (15.29)
No (1593)	567 (35.59)	155 (9.73)	3 (0.19)	88 (5.52)	468 (29.38)	136 (8.54)
MTI prevention	*P* = 0.180	*P* = 0.354	*P* = 0.070	*P* = 0.484	*P* = 0.243	*P* = 0.085
Yes (85)	37 (43.53)	11 (12.94)	1 (1.18)	6 (7.06)	31 (36.47)	12 (14.12)
No (1585)	576 (36.34)	156 (9.84)	3 (0.19)	84 (5.29)	483 (30.47)	137 (8.64)
Clinical signs	*P* < 0.0001	*P* < 0.0001	*P* = 0.022	*P* < 0.0001	*P* < 0.0001	*P* < 0.0001
Yes (722)	384 (53.18)	106 (14.68)	4 (0.55)	57 (7.89)	333 (46.12)	108 (14.96)
No (943)	227 (24.07)	58 (6.15)	0 (0)	31 (3.29)	182 (19.3)	39 (4.13)
Travel history	*P* = 0.631	*P* = 0.680	*P* = 0.124	*P* = 0.453	*P* = 0.572	*P* = 0.742
Yes (331)	118 (35.65)	35 (10.57)	2 (0.60)	15 (4.53)	98 (29.61)	31 (9.36)
No (1365)	506 (37.07)	134 (9.82)	2 (0.15)	76 (5.57)	426 (31.21)	120 (8.79)

*Abbreviations*: *A Anaplasma* spp., *Bb B. burgdorferi* (*sensu lato*), *Di D. immitis*, *Ec E. canis*, *TTI* tick-transmitted infections; *MTI* mosquito-transmitted infections

**Table 7:** Risk factors for positivity to CVBD agents

Dependent variable risk factor	*P*	OR	95 % CI
Positivity			
≥ 1 year	0.011	1.94	1.15–3.29
Medium size	0.003	1.54	1.16–2.07
Large size	0.002	1.42	1.14–1.79
Outdoors	< 0.0001	2.33	1.82–2.98
Tick contact	< 0.0001	7.13	4.89–10.39
TTI prevention	< 0.0001	2.58	1.66–4.03
Clinical signs	< 0.0001	3.58	2.91–4.42
Positivity to *A*			
Outdoors	0.001	2.02	1.31–3.09
Tick contact	< 0.0001	11.93	4.39–32.42
Clinical signs	< 0.0001	2.63	1.88–3.67
Positivity to *Bb*			
Clinical signs	0.022	nd	nd
Positivity to *Di*			
Medium size	0.035	1.83	1.03–3.24
Large size	0.004	2.04	1.24–3.36
Outdoors	< 0.0001	4.12	1.98–8.59
Tick contact	0.002	3.24	1.48–7.08
Clinical signs	< 0.0001	2.52	1.61–3.95
Positivity to *Ec*			
≥ 1 year	0.014	2.02	1.14–3.57
Medium size	0.018	1.44	1.06–1.94
Large size	0.009	1.37	1.08–1.74
Outdoors	< 0.0001	2.21	1.69–2.87
Tick contact	< 0.0001	7.23	4.74–11.04
TTI prevention	< 0.0001	3.27	2.10–5.09
Clinical signs	< 0.0001	3.58	2.88–4.45
Co-infections			
Large size	0.007	1.75	1.16–2.62
Outdoors	< 0.0001	3.06	1.82–5.14
Tick contact	< 0.0001	14.22	4.50–44.91
TTI prevention	0.033	1.93	1.04–3.58
Clinical signs	< 0.0001	4.08	2.79–5.96

*Abbreviations*: *OR* odds ratio, *CI* confidence interval, *nd* no data

## Discussion

**Fig. 2:**
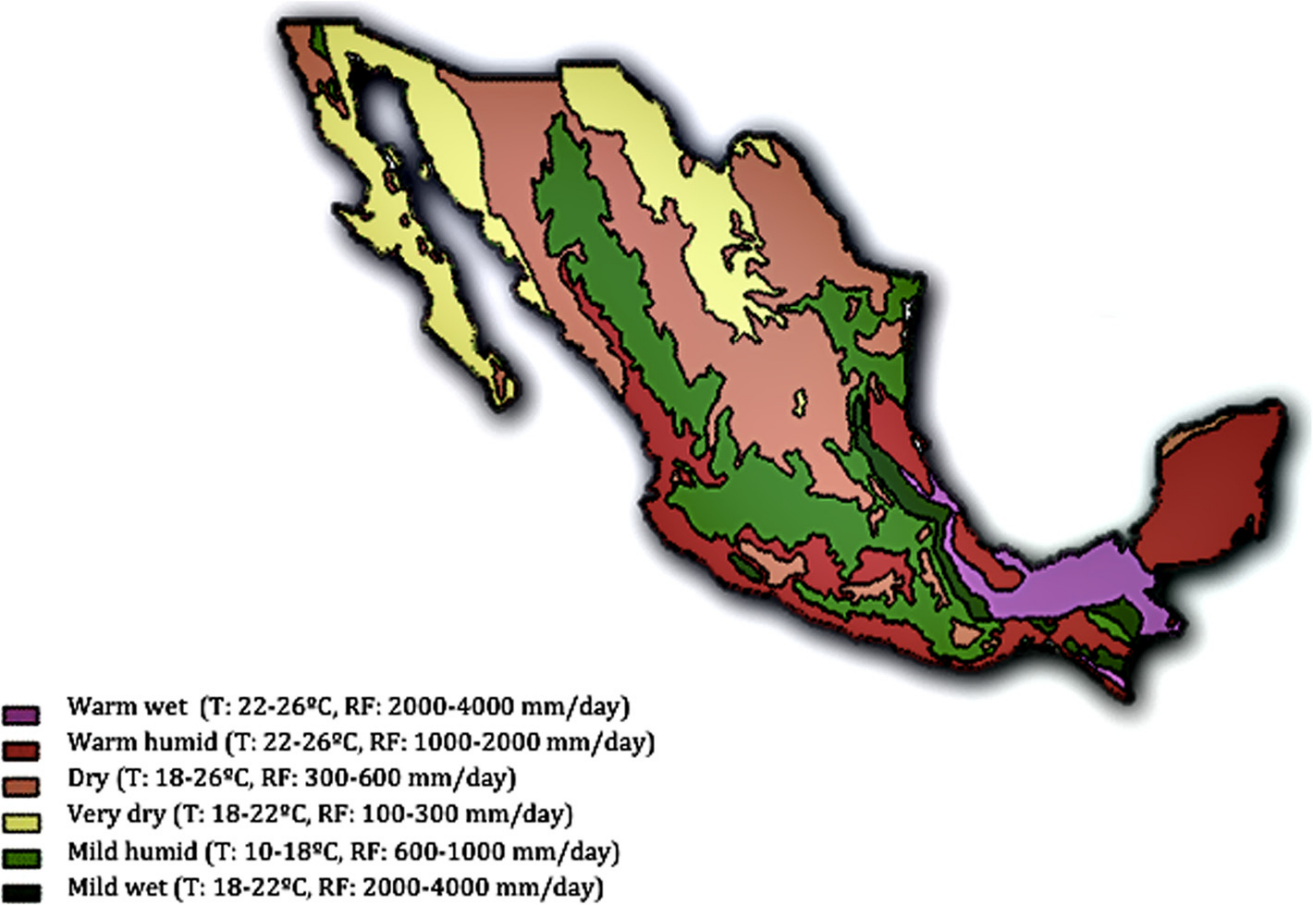
Climatic regions of Mexico. Division of Mexico, according to humidity and temperature, into six different climatic areas with a warm wet, warm humid, dry, very dry, mild humid and mild wet climate, respectively

Countrywide serological evaluation for four CVBD agents was carried out in Mexico. The regions described in the study presented here resulted from a geoeconomic division of the country. This classification is not coincidental with a climatic division, which according to humidity and temperature categorizes the country into areas with a warm wet, warm humid, dry, very dry, mild humid and mild wet climate (Fig. 2) [21].

Seropositivity values for *Anaplasma* spp.*, B. burgdorferi* (*sensu lato*)*, D. immitis* and *E. canis* reported here must be interpreted as current infection with or previous exposure to the pathogens under assessment.

Positive dogs to *Anaplasma* spp. were 9.9 %*,* with the highest prevalence detected in the northwestern (16.4 %) and lowest in the northcentral states of the country (0.6 %). Due to serological cross-reactivity between *A. phagocytophilum* and *A. platys*, results presented here indicated exposure to *Anaplasma* spp., but unfortunately the lack of molecular assessment limited species-level identification. To our knowledge, this is the first study documenting seroprevalence of *Anaplasma* spp. in several states of Mexico. To date only anecdotal reports have documented the presence of *Anaplasma* spp. in this country [31, 51]. Seroprevalence detected in the northeastern region in this study was 6.6 %. This percentage is higher than detected previously by ELISA testing (3 %) among 391 dogs from Monterrey (Nuevo León) [51]. In contrast, the prevalence of infection for *Anaplasma* 16S rRNA obtained by molecular identification in a recent study conducted among 100 healthy dogs infested by ticks, in Coahuila and Durango, was 31 % [31].

The very low *B. burgdorferi* (*sensu lato*) seroprevalence (0.2 %) is one of the more important findings in this study. Each of the four positive dogs was from the eastern, southeastern, northeastern and northwestern region, respectively. Data were not completely recorded for these dogs, so if they were strays or pets that had traveled to Lyme endemic regions, it would not be unexpected to get such a low seroprevalence with a test with excellent specificity [50]. Alternatively, these could be false positives. All of the dogs positive for *B. burgdorferi* (*sensu lato*) presented clinical signs compatible with CVBD, that finding is in agreement with the fact that the SNAP 4DX kit could be more sensitive to detect antibodies during active infection [26], which could not explain the low seroprevalence recorded here. Low prevalence (0.9 %) was previously reported among 338 dogs from Nuevo Leon, detected by PCR assay [29]. In contrast, higher seroprevalence for *B. burgdorferi* (16 %) was detected by indirect immunofluorescence assay among 850 dogs from Monterrey, a city located in the Northeast of Mexico [23]. A previous study conducted in the same area reported the presence of a high proportion of ticks infected with *B. burgdorferi* (*sensu stricto*), supporting that the Northeast should be considered a zone where Lyme disease is endemic [25]. A survey conducted in Mexico City and northeastern regions detected a seroprevalence of 3.43 and 6.2 %, respectively, among 2346 canine serum samples that were analysed by ELISA and subsequently confirmed by Western Blot technique [52]. Higher prevalence (8.2 %), was also detected by ELISA in 94 dogs from Mexicali (Baja California), where the known vectors of this disease were not found [26].

Although little is known about Lyme disease and its transmission cycle in this country, seroprevalence of antibodies to *B. burgdorferi* (*sensu lato*) in dogs could be related to human cases of Lyme disease in Mexico [23]. A previous study concluded that the highest potential for transmission was found in high-altitude and low-temperature regions, where *Ixodes* species are known vectors [28].

Epidemiological data about dirofilariosis in Mexico is very variable and results from previous studies are difficult to compare due to differences in the efficacy of the diverse diagnostic techniques used, size and geographical origin of the population examined and study season [22, 27]. The prevalence of heartworm infection at the national level was previously estimated to be around 7.5 % from Knott and ELISA diagnosis, with highest prevalence (19.6 %) among dogs from coastal areas [24]. In this study, *D. immitis* was detected in 5.3 % of all dogs with the higher percentage of infection (8.9 %) in the notheastern region. *Dirofilaria immitis* was not detected in dogs from the northcentral area, which is in agreement with a previous publication that reported a low proportion of infection (1.3 %) in post-mortem examination of 378 dogs from Queretaro, Central Mexico [27]. Positivity of 7.9 % for *D. immitis* was found in the southeastern region in the present study, which is comparable with previous reported data (8.3 %) in a study conducted in Yucatan among 676 dogs that were evaluated by modified Knott’s and thick drop techniques in blood [53]. However, a study conducted in Celestun, a coastal locality in Southeast Mexico, detected a prevalence of infection as high as 59.8 % among 279 asymptomatic dogs, probably because molecular techniques were used, the widespread distribution of mosquitoes and the presence of other mammals that might contribute to the infection of vectors [54]. The abundance of *Ochlerotatus taeniorhynchus*, a potential main vector in the coastal area, could be the most likely explanation for the difference in dirofilariasis prevalence observed between inland and coastal canine populations [54]. Moreover, a high percentage of co-infection with *Trypanosoma cruzi*, which may interfere significantly with host responses, has been reported in this region, encouraging prophylaxis for dogs [55, 56].

The overall *E. canis* seroprevalence documented here (30.8 %) is in accordance with the results of a previous survey conducted in 25 states of Mexico (33.1 %), which analysed 2395 canine blood samples by ELISA testing [57]. *Ehrlichia canis* accounts in this study for the highest prevalence (51.0 %) in the northwestern region, which is lower than reported before in the State of Sinaloa (74.5 %), although only 152 dogs infested with ticks were evaluated by ELISA testing in that study [58]. The high proportion of infection detected in the southeastern region (35.4 %) is in agreement with previous studies conducted in Yucatan [30, 59]. Reported prevalence among 50 dogs by blood smear evaluation was 4 %, while 36 % of these dogs were positive by PCR testing [30]. In the same study, higher values were found by molecular techniques when only dogs from an animal shelter were evaluated (45 %). It was presumed that those dogs were more likely infested by *R. sanguineus* (*s.l*.) and lived at a lower hygiene level [30]. Another survey conducted in the same area reported similar results among 120 dogs [59]. Analysis of blood samples using ELISA testing and blood smear evaluation showed 5.0 and 44.1 % positivity, respectively [59]. However, results are in contrast to a previous survey performed in Yucatan [60]. That study included 309 stray dogs, which were evaluated by immunofluorescence antibody test in blood and indirect immunoperoxidase technique, reporting 8.7 and 8.1 % prevalence of infection, respectively [60]. A possible explanation could be the diagnostic techniques used that differed greatly from ELISA testing performed in the present study. Seroprevalence in the northeastern region was 22.9 %. A lower prevalence for *E. canis* (4 %) has been previously detected by PCR among 100 healthy dogs infected with ticks, in Coahuila and Durango [31]. This disagreement may be due to the phenomenon black freezing that occurred during the sampling period of that study, which was characterized by an abnormally low average temperature of -5 °C, during four days in February, which decreased the amount of ticks infesting dogs. Another factor could be the different diagnostic techniques used. Additionally, all the dogs included in that study were healthy dogs, which is in contrast with the population included in the present survey [31].

Several epidemiological variables were considered in the present study, in order to evaluate potential associations with seropositivity to CVBD agents. Prevalence encountered for each pathogen varied depending on the geographical region. Despite this finding, dogs with travel history to other regions or abroad did not show significant differences in seroprevalence.

No link was detected between gender and positivity for CVBD agents, which is in agreement with previous studies [38, 43, 46, 59], even though distribution of tick infestation has been reported to be strongly dependent on female dogs because they are more sedentary than males when feeding their puppies [29].

Seropositivity value for *E. canis* in dogs aged one year or older was significantly higher compared with younger dogs. It is coincidental with previous studies that proposed the immunological status of the host or the increasing exposure to the vector with age as possible explanations for this finding [39, 59]. Controversially, a survey performed in Spain showed a higher proportion of infection for *E. canis* and *B. burgdorferi* (*sensu lato*) among dogs aged less than one year compared to older dogs [43]. Comparable to other studies [22, 46], no significant association between age and positivity for the rest of the CVBD agents was detected in this study. By contrast, it has been suggested that the age of dogs could be an important risk factor for filarial infection, because older dogs are exposed for longer time periods to mosquito bites than younger dogs, in endemic areas [39, 56].

As reported previously [22], positivity among small-breed dogs in this study was significantly lower compared to dogs of medium and large size.

Correlation between the presence of clinical signs and seropositivity was statistically significant for all of the CVBD agents analysed. Co-infections were the main cause of illness detected in CVBD-suspect dogs. According with previous publications, the most common combinations included *Anaplasma* spp., *D. immitis* and *E. canis* [39]*.* A previous report documenting high prevalence of *E. canis* infection in Mexico highlighted that almost half of the population remains subclinically infected [57]. Thus, a routine serological evaluation every six months was recommended in dogs presenting or not clinical signs consistent with CVBD [57]. In contrast, no relationship between seropositivity and the sick status of the dogs was found in studies conducted in other countries [35, 46]. It has been reported that clinical signs may vary among and within geographical locations. Proposed reasons include pathogen strain variations, dose of infectious organism, breed of dogs examined, immunological status of the host and concurrent infections with other CVBD agents [59].

As described before [30, 57], results showed that dogs infested with ticks had a higher percentage of seropositivity for all the pathogens under assessment. This conclusion would support the hypothesis that prevalence in dogs living outdoors should be higher compared to indoors, because the increased exposure to the vectors, but no significant association was detected between *B. burgdorferi* (*sensu lato*) infection and the place where dogs lived in this study. However, the low number of positive dogs for borreliosis detected in the study presented here precludes any conclusion.

Effective preventive measures are not always affordable in Mexico, so off-label injectable bovine ivermectin formulation and/or tetracycline, doxycycline and imidocarb are routinely used to treat suspected infections with CVBD agents [24]. The use of ivermectin promotes a reduction in the number of microfilaremic dogs and, therefore, the source of infection for the mosquito population. In addition, tetracyclines are known to destroy intracellular bacteria of the genus *Wolbachia*, a filarial endosymbiont, potentially contributing to a decline heartworm prevalence [24]. In contrast, dogs that received treatment for tick-transmitted infections, such as tetracycline, doxycycline or imidocarb, showed significantly higher seroprevalence for *E. canis,* in this study. Similar association was observed previously in an epidemiological study conducted in Romania [40]. One possible explanation for this finding is that most of the dogs treated for tick-transmitted infections in the study presented here had detectable infestation with ticks (76/85), one of the main risk factors for positivity to CVBD agents. On the other hand, positivity for any CVBD agent was not associated with the use of macrocyclic lactones against canine heartworm infection in the present survey.

One of the limitations of this study is that a geoeconomic division of the country instead of a climatic division was used when it was designed. Discrepancies between both classifications could have influenced the results obtained for different regions depending on the location of the veterinary practices. Furthermore, it is worthy of note that despite the wide geographical distribution of the veterinary centers that participated in this study, a potential bias derived from selection of the cities could have influenced the results obtained here.

## Conclusions

This study adds valuable epidemiological information about the current situation of *Anaplasma* spp., *B. burgdorferi* (*sensu lato*), *D. immitis* and *E. canis* across Mexico. The findings highlight that dogs in Mexico are at risk of acquiring any of the zoonotic CVBD, which were under assessment. Veterinarians must be alert about this situation as they have a key role in the identification and control of these diseases in dogs and humans. Nowadays, updated information and products are widely available, which allows provision of better and more effective diagnostic and preventive programmes for dogs.

## Abbreviations

*A*, *Anaplasma* spp.; *Bb*, *Borrelia burgdorferi* (*sensu lato*); CI, confidence interval; CVBD, canine vector-borne disease; *Di*, *Dirofilaria immitis*; *Ec*, *Ehrlichia canis*; ELISA, enzyme-linked immunosorbent assay; MTI, mosquito-transmitted infections; nd, no data; OR, odds ratio; TTI, tick-transmitted infections

## Additional file

Additional file 1: Dataset supporting the conclusions of this article. (XLSX 227 kb)
